# Stillbirth surveillance consortium

**DOI:** 10.1186/1471-2393-15-S1-A8

**Published:** 2015-04-15

**Authors:** Paul A Romitti

**Affiliations:** 1 Department of Epidemiology, College of Public Health, The University of Iowa, Iowa City, Iowa, USA

## 

Stillbirths have an estimated prevalence of 6 per 1,000 live births and fetal deaths combined in the United States (U.S.), yet major risk factors for these adverse birth outcomes remain elusive [1,2]. Historically in the U.S., surveillance information about stillbirth has come from fetal death certificates maintained by the National Vital Statistics System.

Data collection within the National Vital Statistics System is guided by the Model State Vital Statistics Act and Regulations (Model Law), which defines fetal loss as showing no signs of breath or cardiac activity after expulsion [3]. The Model Law also recommends reporting a fetal loss as a stillbirth if the fetus weighs 350 grams or greater, or if no birth weight is available, the fetus is at least 20 weeks in gestational age.

Each state in the U.S. develops its own definition, reporting criteria, and fetal death certificate, which can produce variability in reporting across states [4]. Additionally, previous studies suggest that fetal death certificate data are limited in utility as a source for national stillbirth surveillance due to under- or over-reporting and the completeness and quality of recorded data [5].

An alternative approach to surveillance of stillbirths is the use of established birth defect surveillance systems to incorporate active case finding for stillbirths. This approach permits population-based identification of affected pregnancies and characterization of the epidemiology of these pregnancies. In 2005, the Centers for Disease Control and Prevention established projects for stillbirth surveillance using the established infrastructures of the Metropolitan Atlanta Congenital Defects Program and the Iowa Registry for Congenital and Inherited Disorders, two premier birth defect surveillance systems in the U.S.

The goals of our Iowa project, the Iowa Stillbirth Surveillance Project (ISSP), were to: evaluate the feasibility of expanding the Iowa Registry for Congenital and Inherited Disorders to incorporate data from existing records on stillbirths; monitor and report, as feasible, on the occurrence of stillbirths in the state of Iowa; serve as a registry for etiologic studies of stillbirths; and serve as a resource for education and evaluation of prevention programs that aim to reduce the occurrence of stillbirths .

Active case finding and record abstraction approaches, originally developed for birth defect surveillance in Iowa, were used by the ISSP for state-wide ascertainment of stillbirths. We ascertained 1,301 reportable stillbirths (≥20 weeks gestation or ≥350 grams delivery weight) delivered from January 1, 2005 through December 31, 2011. Surveillance data collected are being used to estimate population-based prevalence estimates for stillbirths in Iowa and to examine fetal and parental characteristics associated with stillbirths. Also, these data are being used to conduct individual-level geospatial surveillance of stillbirths. Knowledge of the spatial patterns of stillbirths may provide important insights into possible links to environmental exposures and the opportunity to plan detailed etiological investigations.

We continue to monitor stillbirths among the approximately 40,000 deliveries in Iowa annually. In 2012, we also expanded active case finding and record abstraction for stillbirths to include birth defect surveillance systems in Colorado, Hawaii, and New York State. This Stillbirth Surveillance Consortium (SSC) uses similar surveillance methods and tools to provide a systematic approach to population-based surveillance of stillbirths and covers more than 120,000 deliveries annually with a diverse racial/ethnic composition. To date, the SSC has ascertained 784 reportable stillbirths delivered from January 1, 2010 through December 31, 2011. Surveillance data collected by the SSC will expand ongoing analyses by the ISSP for prevalence estimation, examination of fetal and parental characteristics, and individual-level geospatial surveillance. The methods developed by the SSC can serve as a model for other states to expand birth defect surveillance programs to include active case finding and record abstraction for stillbirths.
